# Critical Assessment of the Important Residues Involved in the Dimerization and Catalysis of MERS Coronavirus Main Protease

**DOI:** 10.1371/journal.pone.0144865

**Published:** 2015-12-14

**Authors:** Bo-Lin Ho, Shu-Chun Cheng, Lin Shi, Ting-Yun Wang, Kuan-I Ho, Chi-Yuan Chou

**Affiliations:** Department of Life Sciences and Institute of Genome Sciences, National Yang-Ming University, Taipei 112, Taiwan; Saint Louis University, UNITED STATES

## Abstract

**Background:**

A highly pathogenic human coronavirus (CoV), Middle East respiratory syndrome coronavirus (MERS-CoV), has emerged in Jeddah and other places in Saudi Arabia, and has quickly spread to European and Asian countries since September 2012. Up to the 1^st^ October 2015 it has infected at least 1593 people with a global fatality rate of about 35%. Studies to understand the virus are necessary and urgent. In the present study, MERS-CoV main protease (M^pro^) is expressed; the dimerization of the protein and its relationship to catalysis are investigated.

**Methods and Results:**

The crystal structure of MERS-CoV M^pro^ indicates that it shares a similar scaffold to that of other coronaviral M^pro^ and consists of chymotrypsin-like domains I and II and a helical domain III of five helices. Analytical ultracentrifugation analysis demonstrated that MERS-CoV M^pro^ undergoes a monomer to dimer conversion in the presence of a peptide substrate. Glu169 is a key residue and plays a dual role in both dimerization and catalysis. The mutagenesis of other residues found on the dimerization interface indicate that dimerization of MERS-CoV M^pro^ is required for its catalytic activity. One mutation, M298R, resulted in a stable dimer with a higher level of proteolytic activity than the wild-type enzyme.

**Conclusions:**

MERS-CoV M^pro^ shows substrate-induced dimerization and potent proteolytic activity. A critical assessment of the residues important to these processes provides insights into the correlation between dimerization and catalysis within the coronaviral M^pro^ family.

## Introduction

A highly pathogenic human coronavirus (CoV)^1^, Middle East respiratory syndrome coronavirus (MERS-CoV), emerged in Jeddah and other places in Saudi Arabia in September 2012 and spread to some European, African and Asian countries in recent years [1–3]. The virus causes symptoms similar to severe acute respiratory syndrome coronavirus (SARS-CoV), but an additional component is also involved, namely acute renal failure [4]. Up to the 1^st^ October 2015, 1593 people had been infected with MERS and this has led to 568 reported deaths globally (World Health Organization, global alert and response, http://www.who.int/csr/don/01-october-2015-mers-jordan/en/). A serological study of major livestock in Saudi Arabia suggested dromedary camels may be an original reservoir [5, 6]; although recent studies have identified bats may also as the suspected host as a number of bat CoVs show high sequence similarity to SARS-CoV and MERS-CoV [7, 8] and a bat-CoV was discovered that readily infect human cells using ACE2 as the receptor [9]. Nevertheless, human-to-human transmission of MERS-CoV has been confirmed. This May, an infected traveler from the Middle East region returned to his home country of the Republic of Korea and caused an outbreak that revolved around health facilities (http://www.who.int/csr/don/07-july-2015-mers-korea/en/). These findings indicate that it is possible for the virus to spread globally and as such it poses a significant threat to world health and the world economy in general. Therefore studies that help to understand this virus and aid the development of antiviral drugs or other therapies are important.

Similar to other CoVs, after entering the host cells, the nonstructural polyproteins (pp1a and pp1ab) of MERS-CoV are synthesized and then cleaved by two coronaviral proteases, a main protease (M^pro^) (EC 3.4.22.69) and a papain-like protease (EC 3.4.22.46) [10]. This cleavage is considered to be a leading process which is required for viral maturation [11–14]. The MERS-CoV M^pro^, namely nsp5 of the pp1a proteins (residue 3248–3553), has been identified [15]. Like other M^pro^, there is a catalytic dyad that consists of a His residue and a Cys residue [16–20]. Sequence alignment suggests that MERS-CoV M^pro^, in a similar manner to other known M^pro^, has a chymotrypsin-like architecture consisting of a catalytic core (domain I and II) and a helical domain III; sequence identities of CoV M^pro^ protein range from 50% to 80% (S1 Fig). Recently the crystal structure of an inactive MERS-CoV M^pro^ C148A mutant has confirmed this similarity and the results also suggest that this protein forms a dimer [15]. Furthermore, based on the identification of eleven canonical cleavage sites, the MERS-CoV M^pro^ should be able to recognize and cleave at the (L/M)-Q-↓-(A/S) conserved sequence, which is essential for most CoV M^pro^-mediated processing [21, 22]. However, up to now, the correlation between the protein’s structure and the catalytic process remains unclear.

In the present study we expressed and purified the MERS-CoV M^pro^ using the authentic N-terminus via an *Escherichia coli* system. The crystal structure of the MERS-CoV M^pro^ at 3.0-Å resolution is reported. The quaternary structural changes in the MERS-CoV M^pro^ in the absence and presence of peptide substrates were investigated by analytical ultracentrifugation (AUC). The results of kinetic activity assays indicated that MERS-CoV M^pro^ exhibits potent proteolytic activity that is associated with a pattern of cooperativity. Some critical residues for dimerization of the protein and catalysis by the protein were verified by site-directed mutagenesis. The present studies provide a foundation for an understanding of the mechanism that controls the monomer-dimer switch at work in MERS-CoV M^pro^.

## Materials and Methods

### Expression Plasmid Construction

The sequence of the MERS-CoV M^pro^ (GenBank accession number AHC74086; polyprotein residues 3248–3553) was synthesized (MDBio Inc.), digested by *Nde*I-*Xho*I and then inserted into the vector pET-28a(+) (Novagen). In this construct, the 6 x His tag is retained at the N-terminus. To remove the fusion tag and generate an authentic N-terminus for protein purification, the codons of the thrombin cutting recognition sequence and a *Nde*I cutting site were removed and then inserted the codons of Leu-Arg-Leu-Lys-Gly-Gly into the above vector. The forward primer sequence for site-directed mutagenesis was 5’-CATCACAGCAGCGGCCTGCGTCTGAAAGGCGGCAGCGGTTTGGTGAAAATG-3’ and the reverse primer was 5’-CATTTTCACCAAACCGCTGCCGCCTTTC AGACGCAGGCCGCTGCTGTGATG-3’. The reading frame of the final plasmid was confirmed by sequencing.

### Expression and Purification of MERS-CoV M^pro^

The expression vector was transformed into *E*. *coli* BL21 (DE3) cells (Novagen). Cultures were grown in 0.8 liters of LB medium at 37°C for 4 h, induced with 0.4 mM isopropyl-β-_D_-thiogalactopyranoside, and then incubated overnight at 20°C. After centrifuging at 6,000 x g at 4°C for 15 min, the cell pellets were resuspended in lysis buffer (20 mM Tris, pH 8.5, 250 mM NaCl, 5% glycerol, 0.2% Triton X-100, and 2 mM β-mercaptoethanol) and then lysed by sonication. The crude extract was then centrifuged at 12,000 x g at 4°C for 25 min to remove the insoluble pellet. Next the supernatant was incubated with 1-ml Ni-NTA beads at 4°C for 1 h and then loaded onto an empty column. After allowing the supernatant to flow through, the beads were washed with washing buffer (20 mM Tris, pH 8.5, 250 mM NaCl, 8 mM imidazole, and 2 mM β-mercaptoethanol). The SARS-CoV papain-like protease [12] (1 mg in 100 mM phosphate buffer (pH 6.5)) was then added and incubated for 3 h. The SARS-CoV papain-like protease digestion, which removed the 6 x His tag and Leu-Arg-Leu-Lys-Gly-Gly fragment, resulted in a native protein product with an authentic N-terminus. The digest was allowed to flow through and then loaded onto a S-100 gel-filtration column (GE Healthcare) equilibrated with running buffer (20 mM Tris, pH 8.5, 100 mM NaCl, and 2 mM dithiothreitol). The purity of the fractions collected was analyzed by SDS-PAGE and the protein was concentrated to 30 mg/ml by Amicon Ultra-4 10-kDa centrifugal filter (Millipore).

### Protein Crystallography

Crystals of the MERS-CoV M^pro^ were obtained at 295 K by the sitting-drop vapor-diffusion method. The protein solution was set up at 5 mg/ml and the reservoir solution consisted of 0.1 M Tris, pH 8.4, 15% (w/v) PEG 4000 and 0.2 M sodium acetate. Clusters of needle crystals appeared in 2 days and were used for micro-seeding. Single cystals of rectangle shape and with dimensions of 0.3–0.5 mm were obtained in less than a week. All crystals were cryoprotected in the reservoir solution with 15% glycerol and were flash-cooled in liquid nitrogen.

### Data collection, structure determination and refinement

X-ray diffraction data were collected at 100 K on the SPXF beamline 13C1 at the National Synchrotron Radiation Research Center, Taiwan, ROC, using a ADSC Quantum-315r CCD detector (X-ray wavelength of 0.976 Å). The diffraction images were processed and scaled using the HKL-2000 package [23]. The structure was solved by the molecular replacement method by Phaser [24] using the structure of SARS-CoV M^pro^ R298A mutant (PDB entry 4hi3; [25]) as the search model. Manual rebuilding of the structure model was performed using Coot [26]. Structure refinement was carried out using REFMAC [27]. The data-processing and refinement has been deposited in the Protein Data Bank (PDB entry 5c3n).

### Steady-State Kinetic Analysis

The colorimetry-based peptide substrate, TSAVLQ-para-nitroanilide (TQ6-pNA) (purity 95–99% by HPLC; GL Biochem Ltd, Shanghai, China), was used to measure the proteolytic activity of MERS-CoV M^pro^ and its mutants throughout the course of the study as described previously [25, 28]. This substrate is cleaved at the Gln-pNA bond to release free pNA, resulting in an increase in absorbance at 405 nm. The absorbance at 405 nm was continuously monitored using a Jasco V-550 UV/VIS spectrophotometer. The protease activity assay was performed in 10 mM phosphate (pH 7.6) at 30°C. The substrate stock solution was 1600 μM and the working concentrations were from 25 to 1200 μM. In the substrate titration assay, the concentration of MERS-CoV M^pro^ and its mutants, V4R, T126S, E169A, M298R and T126S/M298R was 0.3, 0.4, 0.7, 1.2, 0.15 and 0.26 μM, respectively, while that of SARS-CoV M^pro^ was 1.1 μM. Steady state enzyme kinetic parameters were obtained by fitting the initial velocity (ν_0_) data to the Michaelis-Menten Eq (1)

$$\nu_{0}=\frac{{\text{k}}_{\text{cat}}\left[\text{E}\right]\left[\text{S}\right]}{{\text{K}}_{\text{m}}+\left[\text{S}\right]}$$ (1)

where k_cat_ is the catalytic constant, [E] is the enzyme concentration, [S] is the substrate concentration and K_m_ is the Michaelis constant of the substrate. The program SigmaPlot (Systat Software, Inc., Richmond, CA) was used for the data analysis.

To assess the cooperativity effect, the kinetic parameters were obtained by fitting the initial velocities to the Hill Eq (2)

$$\nu_{0}=\frac{{\text{k}}_{\text{cat}}\left[\text{E}\right]{\left[\text{S}\right]}^{h}}{\text{K'}+{\left[\text{S}\right]}^{h}}$$ (2)

where K’ is a constant that is related to the dissociation constant and *h* is the Hill constant.

### Analytical ultracentrifugation analysis

AUC was performed on a XL-A analytical ultracentrifuge (Beckman Coulter) using an An-50 Ti rotor [11, 12, 25, 28–30]. The sedimentation velocity experiments were carried out using a double-sector *epon* charcoal-filled centerpiece at 20°C with a rotor speed of 42,000 rpm. Protein solutions of 0.05 to 0.5 mg/ml (330 μl) and reference (370 μl) solutions, both containing D_2_O, were loaded into the centerpiece. The absorbance at 280 nm was monitored in a continuous mode with a time interval of 300 s and a step size of 0.003 cm. Multiple scans at different time intervals were then fitted to a continuous c(s) distribution model using the SEDFIT program [31]. Additionally, the results with the various different protein concentrations were globally fitted to a monomer-dimer self-association model using the SEDPHAT program to calculate the dissociation constant (K_d_) [32].

To measure the substrate-induced dimerization, the active enzyme centrifugation (AEC) [33] was performed. Briefly, MERS-CoV M^pro^ of 15 μl (1 mg/ml) was added into the small well of the band-forming centerpiece before the cell assembled. Then 330 μl of peptide substrate at 0, 200 and 400 μM in D_2_O were respectively loaded into the bulk sample sector space. At a rotor speed of 42,000 rpm, the protein solution flowed into the substrate-containing channel and form a protein band. It can be detected by absorbance at 250 nm. During the centrifugation, the sediment protein continuously met and cleaved the substrate, which can be detected by absorbance change at 405 nm. The dataset from the multiple scans at 250 nm at various time intervals were fitted to a continuous c(s) distribution model using the SEDFIT program [31], while the first five scans (0–30 min) at 405 nm were used to derive the product concentration and then initial velocity values.

### Isothermal titration calorimetry (ITC)

The protocol followed that of previous studies [28] with some modifications. Apparent dissociation constants and stoichiometry of the enzyme-ligand interactions were measured by a Thermal Activity Monitor 2277 from TA instruments (New Castle, DE). Calorimetric titrations of the peptide substrate TQ6-pNA (0.5 mM in a 250-μl syringe) and M^pro^ (6 μM in a 4-ml ampoule) were carried out at 25°C in 10 mM phosphate buffer (pH 7.6). The peptides were titrated into the enzyme using a 10-μl aliquot for each injection with a time interval of 20 min. A control experiment in the absence of enzyme was performed in parallel to correct for the dilution of heat. The data obtained was then analyzed by integrating the heat effects normalized against the amount of injected protein using curve-fitting based on a 1:1 binding model. This involved the use of Digitam software (TA instruments, New Castle, DE).

## Results and Discussion

### Recombinant MERS-CoV M^pro^ preparation

As part of the present study, an expression vector was constructed and the BL21 (DE3) STAR (Invitrogen) strain of *E*. *coli* were used to express MERS-CoV M^pro^. Unlike SARS-CoV M^pro^ [25, 28], the MERS-CoV M^pro^ with 6 x His-tag retained at the C-terminus cannot be expressed. Instead, the bacteria are able to express the M^pro^ when there is a N-terminal 6 x His-tag fusion that can be removed during the purification. However, thrombin digestion leaves two extra residues (Gly-Ser) at the N-terminus of M^pro^, resulting in protein with no proteolytic activity (data not shown). Therefore we used SARS-CoV papain-like protease [12, 30, 34], which is a highly active viral deubiquitinase and does not leave any residues at the N-terminus of M^pro^. After gel-filtration, the purity of authentic N-terminus M^pro^ was about 99% (S2 Fig). The size of the MERS-CoV M^pro^ was found to be close to 30 kDa, while any uncut protein was located at higher molecular weight position. The typical yield was about 10 mg after purification from 0.8 liter of *E*. *coli* culture.

### Overall structure of MERS-CoV M^pro^

**Fig 1:**
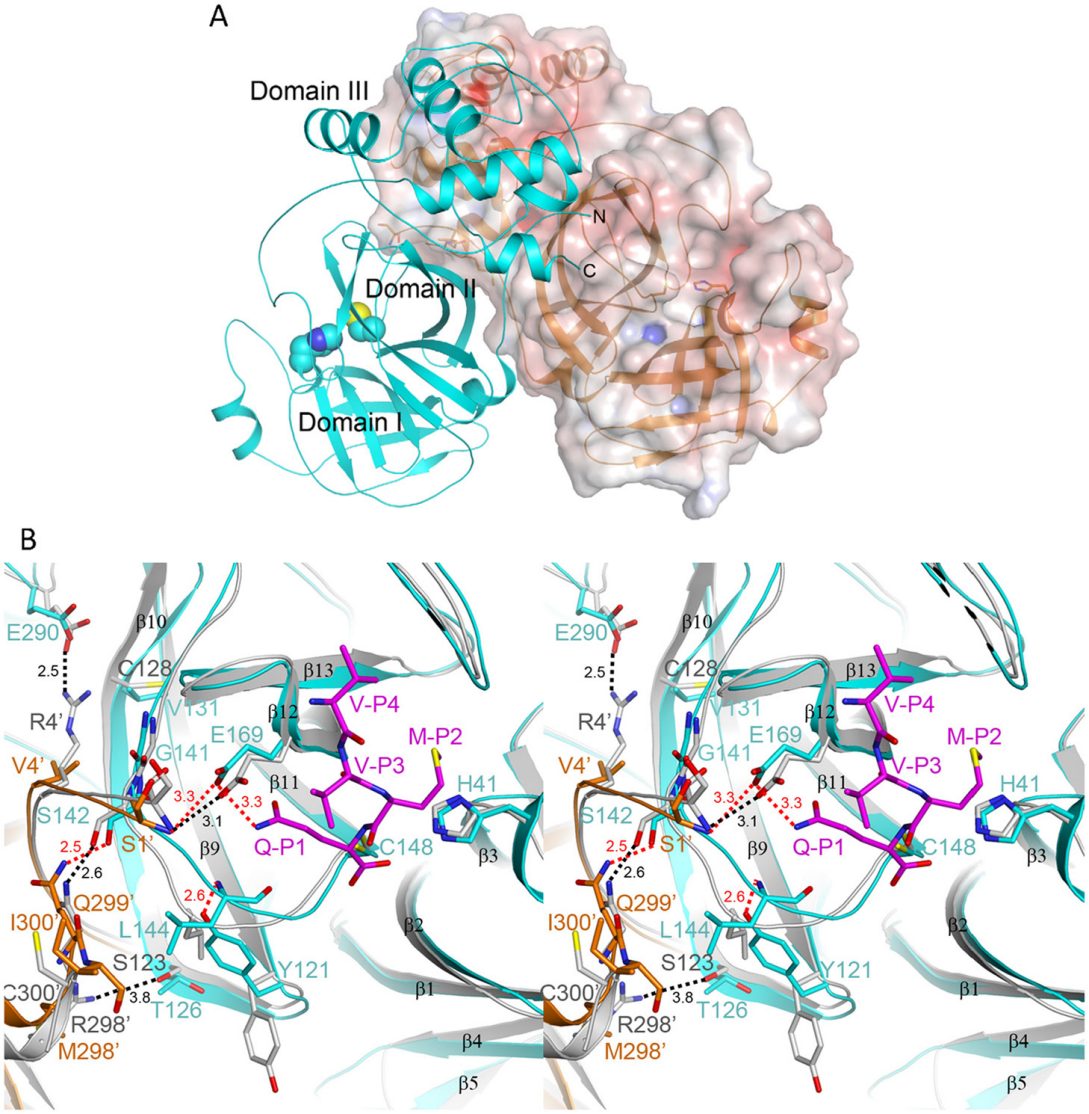
The structure of MERS-CoV M^pro^. (A) The overall structure of the dimeric M^pro^. The two protomers are shown as a ribbon and as a surface model, respectively. The negatively and positively charged regions on the molecular surfaces are colored red and blue. The spheres indicate the catalytic dyad, His41-Cys148. (B) Stereo view of an overlay of the dimerization interface and the active site of MERS-CoV M^pro^ (in cyan and orange) with that of SARS-CoV M^pro^ (grey; [16]). The red dashed lines indicate polar interactions between the two protomers of MERS-CoV M^pro^, while the black dashed lines show polar interactions between those of SARS-CoV M^pro^. The C atoms of the modeled substrate P4-P1 residues (from the structure of C148A mutant [15]) are colored magenta. The structural figures in this paper were produced using PyMol (http://www.pymol.org/).

The structure of the MERS-CoV M^pro^ was determined at 3.0 Å resolution by X-ray crystallography (Table 1 and Fig 1A). The crystal packing belonged to space group *C*222_1_, with unit-cell parameters a = 87.2, b = 94.0, c = 155.1 Å and α = β = γ = 90°. The final atomic model containing two M^pro^ molecules in a crystallographic asymmetric unit agrees well with the crystallographic data and the expected values of geometric parameters (Table 1). There are no residues in the disallowed region of the Ramachandran plot, while 81.3% of the residues are in the most favored region.

**Table 1:** Summary of crystallographic information for MERS-CoV M^pro^.

Data Collection	
Space group	*C*222_1_
Cell dimensions	
*a*, *b*, *c* (Å)	87.2, 94.0, 155.1
α, β, γ (°)	90, 90, 90
Resolution^a^ (Å)	30–3.0 (3.11–3.0)
*R*_merge_ ^b^ (%)	17 (62.9)
*I* / *σI*	10.2 (3.1)
Completeness (%)	94.6 (95.2)
Redundancy	5.5 (5.6)
**Refinement**	
Number of reflections	11,675 (1,549)
*R* factor^c^ (%)	21.8
Free *R* factor^d^ (%)	28.2
Number of atoms	
Protein	4,580
Water	0
Average *B*-factors for protein atoms (Å^2^)	81.6
R.m.s. deviations	
Bond length (Å)	0.009
Bond angles (°)	1.5
Ramachandran plot statistics (%)	
Most favored region	81.3
Additional allowed region	13.6
Generously allowed region	5.2
Disallowed region	0

^a^ The numbers in parentheses are for the highest-resolution shell.

^b^ $R_{merge}=\sum_{h}\sum_{i}|I_{hi}-\langle I_{h}\rangle |/\sum_{h}\sum_{i}I_{hi}$, where *I*_*hi*_ is the integrated intensity of a given reflection and 〈*I*_*h*_〉 is the mean intensity of multiple corresponding symmetry-related reflections.

^c^ $R=\sum_{h}|F_{h}^{o}-F_{h}^{c}|/\sum_{h}F_{h}^{o}$, where $F_{h}^{o}$ and $F_{h}^{c}$ are the observed and calculated structure factors, respectively.

^d^ Free *R* is *R* calculated using a random 5% of data excluded from the refinement.

The overall dimeric structure of M^pro^ is similar to that of SARS-CoV M^pro^; although a relative shift of 10° to 30° could be observed for the two domain III within the dimer (S3A Fig). Indeed, the r.m.s. distance between equivalent Cα atoms of the domain I+II of the two structures is 0.9 Å, while that between the domain III of the two structures is 3.1 Å. Compared with the structures of the ligand-bound complex (PDB entry 4YLU) and C148A mutant (PDB entry 4WME) [14, 15], the r. m. s. distance is 0.8 and 0.7 Å over 540 Cα atom pairs, respectively (S3B Fig). This indicates that the dimeric structures show no significant difference; although the present structure is a free enzyme and the other two structures involve enzyme-ligand complexes and higher resolution. Besides, there is minor difference between the present structure and bat-CoV HKU4 M^pro^ (PDB entry 2YNA), with 80% sequence identity, as the r. m. s. distance over 539 Cα atom pairs is 0.8 Å (S3B Fig).

Interestingly, the dimerization interface situation with the MERS-CoV M^pro^ was found to be different to that of SARS-CoV M^pro^ where there are four amino acid pairs with intermolecular polar interactions (Ser1…Glu166, Arg4…Glu290, Ser123…Arg298 and Ser139…Gln299). There are only two pairs of intermolecular hydrogen bonds, Ser1…Glu169 and Ser142…Gln299 that are associated with the dimer surface of MERS-CoV M^pro^ according to the current structure (Fig 1B). This led us to compare the dimerization and catalytic activity of the two types of M^pro^. In the present study, in addition to using the wild-type MERS-CoV M^pro^, we also mutated several residues at the dimerization interface in order to evaluate their role in dimerization and catalysis of MERS-CoV M^pro^ (see below).

### Correlation between dimerization and catalysis of MERS-CoV M^pro^

**Fig 2:**
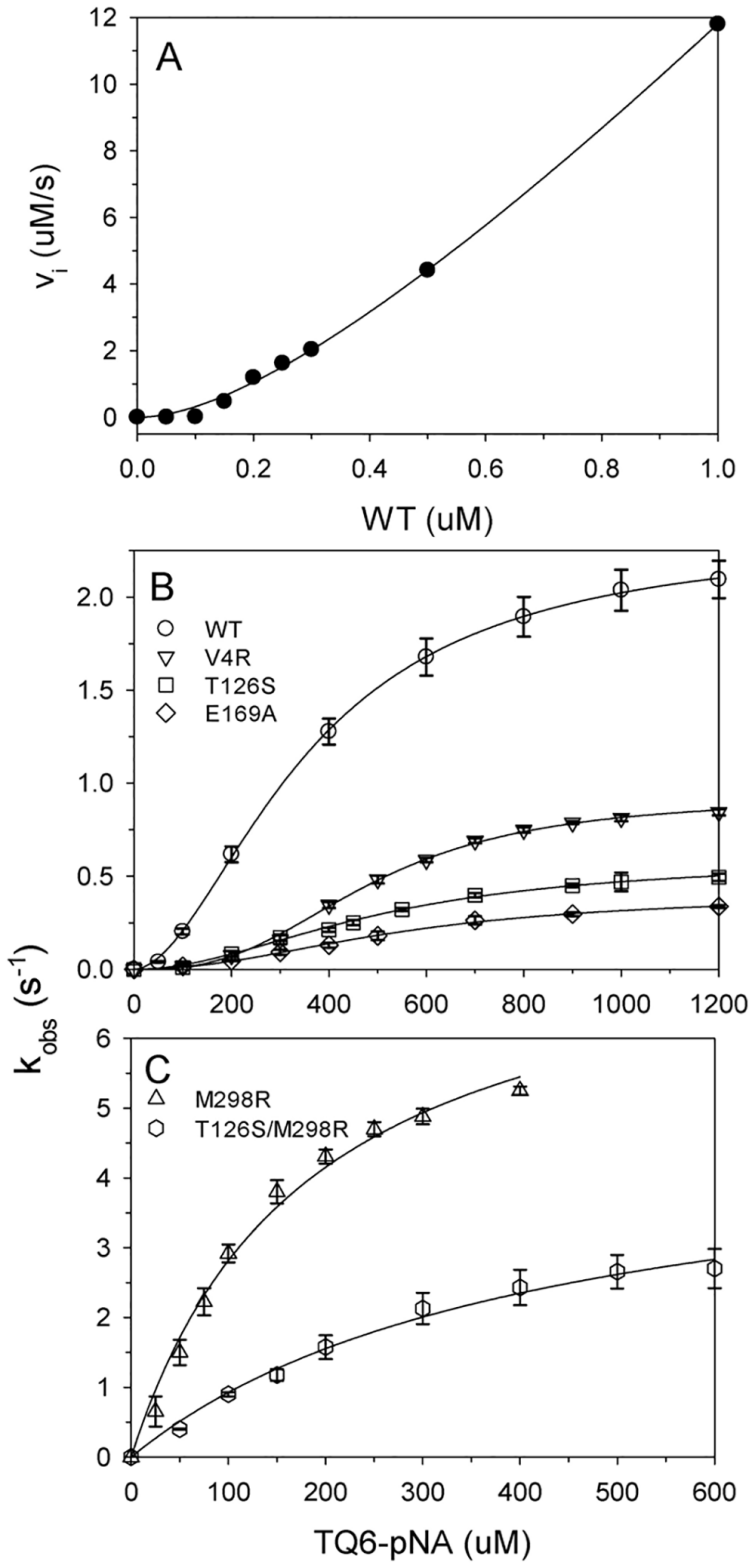
Proteolytic activity assay of MERS-CoV M^pro^ and its mutants. (A) Plot of initial velocities (v_i_) versus the concentration of wild-type MERS-CoV M^pro^. The concentration of substrate (TQ6-pNA) was 600 μM. The line represented the best-fit results according to the nonlinear dependence equation [28]. (B) and (C) Plots of rate constant (k_obs_) versus the concentration of peptide substrate for MERS-CoV M^pro^ (by circles), the V4R mutant (by lower triangles), the T126S mutant (by squares), the E169A (by diamonds), the M298R (by upper triangles) and the T126S/M298R (by hexagons) are indicated. The lines represented the best-fit results according to the Hill equation (Eq 2; wild-type, V4R, T126S and E169A mutants) or the Michaelis-Menten equation (Eq 1; M298R and T126S/M298R mutants). The protein concentrations of the wild-type, V4R, T126S, E169A, M298R and T126S/M298R mutants used for the assay were 0.3, 0.4, 0.7, 1.2, 0.15 and 0.26 μM, respectively. All the assays were performed in 10 mM phosphate (pH7.6) at 30°C and repeated twice to ensure reproducibility and the error bars were shown. The kinetic parameters are shown in Table 2.

To compare catalysis between the two M^pro^, TQ6-pNA, a peptide substrate for SARS-CoV M^pro^ [25, 28], was used to measure the proteolytic activity. At first, the dependence of the initial velocity on enzyme concentration was analyzed and showed a nonlinear upward correlation (Fig 2A). The pattern is similar to that of SARS-CoV M^pro^, as the monomeric M^pro^ may not have catalytic activity [28]. However, MERS-CoV M^pro^ displayed a sigmoid curve for its rate constant pattern at various substrate concentrations (Fig 2B, open circles); this contrast with SARS-CoV M^pro^, which exhibited a classical saturation curve (S4 Fig). The results were then fitted to the Hill equation (Eq 2) in order to evaluate the kinetic parameters (Table 2). The k_cat_ (2.33 s^-1^) of MERS-CoV M^pro^ is close to that of SARS-CoV M^pro^ (2.11 s^-1^), while the Hill constant was 1.8, suggesting a significant degree of positive cooperativity among the M^pro^ protomers. The comparable activity levels of the two M^pro^ in the present study is dissimilar to the results obtained during a recent study in which the activity of the MERS-CoV M^pro^ was found to be 5-fold lower than that of SARS-CoV [14]. Using different substrates may cause the difference. Tomar *et al*. [14] used a longer peptide substrate with residues present at both P and P’ site. However, in the present studies we used a peptide substrate that contains only P site residues. Besides, they utilized FRET substrate and could only be used at low substrate concentrations to prevent the inner-filter effect, while we are able to use higher substrate concentrations to capture the kinetic parameters.

**Table 2:** The kinetic parameters and dissociation constants of MERS-CoV M^pro^.

Proteins	Kinetic Parameters^a^			Dissociation Constant^b^	
	K_m_ (μM)	*k*_cat_ (s^-1^)	*h*	No substrate(μM)	With 600 μM substrate (μM)
MERS-CoV M^pro^					
Wild-type	-	2.33 ± 0.13	1.8 ± 0.04	7.7 ± 0.3	0.7 ± 0.04
V4R	-	0.96 ± 0.05	2.7 ± 0.2	23.0 ± 0.4	15.2 ± 0.3
T126S	-	0.56 ± 0.04	2.0 ± 0.2	33.7 ± 0.9	13.9 ± 0.1
E169A	-	0.41 ± 0.02	2.1 ± 0.1	14.3 ± 0.2	14.1 ± 0.5
M298R	181.0 ± 24.0	7.91 ± 0.49	-	1.1 ± 0.1	0.7 ± 0.01
T126S/M298R	419.4 ± 63.9	4.63 ± 0.37	-	2.8 ± 0.1	0.9 ± 0.01
SARS-CoV Mpro	890 ± 130	2.11 ± 0.15	-	0.7 ± 0.02	1.7 ± 0.03^c^

^a^ Kinetic data of SARS-CoV M^pro^ and MERS-CoV M298R and T126S/M298R mutants were fitted to the Michaelis-Menten equation (Eq 1), while those of the others were fitted to the Hill equation (Eq 2). The R_sqr_ were from 0.985 to 0.999, respectively. All the assays were repeated twice and the average values were used for the fitting.

^b^ The values were derived from a global fit of the AUC data to a monomer-dimer self-association model by SEDPHAT [32]. The experiments for the assay were obtained at protein concentration of 1.5 to 30 μM.

^c^ The value was from our previous studies for comparison [28].

**Fig 3:**
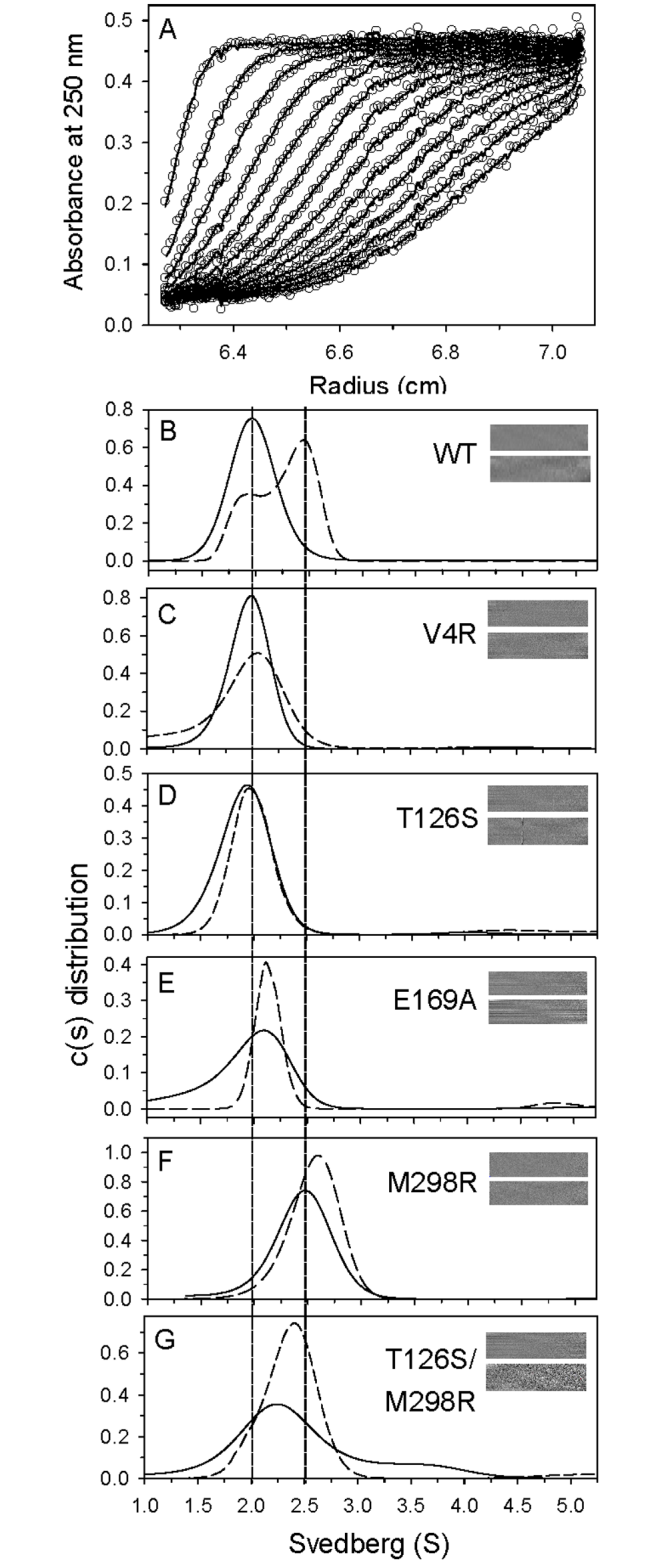
The continuous size distribution change of MERS-CoV M^pro^. (A) Typical trace of absorbance at 250 nm of the enzyme during the SV experiment. The protein concentration was 0.5 mg/ml. For clarity, only every fifth scan is shown. The symbols represent the experimental data and the lines represent the results fitted to the Lamm equation using the SEDFIT program [31]. (B-G) Continuous c(s) distribution of wild-type, V4R, T126S, E169A, M298R and T126S/M298R mutants. The distributions in D_2_O containing 10 mM phosphate (pH 7.6) are shown by solid lines and those in the same buffer but with 600 μM TQ6-pNA substrate are shown by dashed lines. The left vertical dotted line indicates the monomer position and the right dotted line represents the dimer position. The residual bitmap of the raw data and the best-fit results are shown in the insets.

The cooperativity phenomenon associated with the MERS-CoV M^pro^ is similar to that of the SARS-CoV M^pro^ R298A/L monomer mutants; these were found to show monomer to dimer conversion during catalysis [28]. As a result of the above, we investigated the quaternary structure of the M^pro^ by AUC (Fig 3). The cumulative spectra (Fig 3A) were analyzed using the continuous c(s) distribution model and the results suggested that MERS-CoV M^pro^ is a monomer in phosphate buffer (S5 Fig) and this contrasts with a distribution of 30% monomer and 70% dimer in the presence of 600 μM TQ6-pNA (Fig 3B). We also measured the size distribution of M^pro^ at various TQ6-pNA concentrations (S6 Fig). The results indicated that the sedimentation coefficient of the major species was shifted as the substrate dosage changed (S6B Fig). More substrate led to the major species moving close to the dimer position.

**Fig 4:**
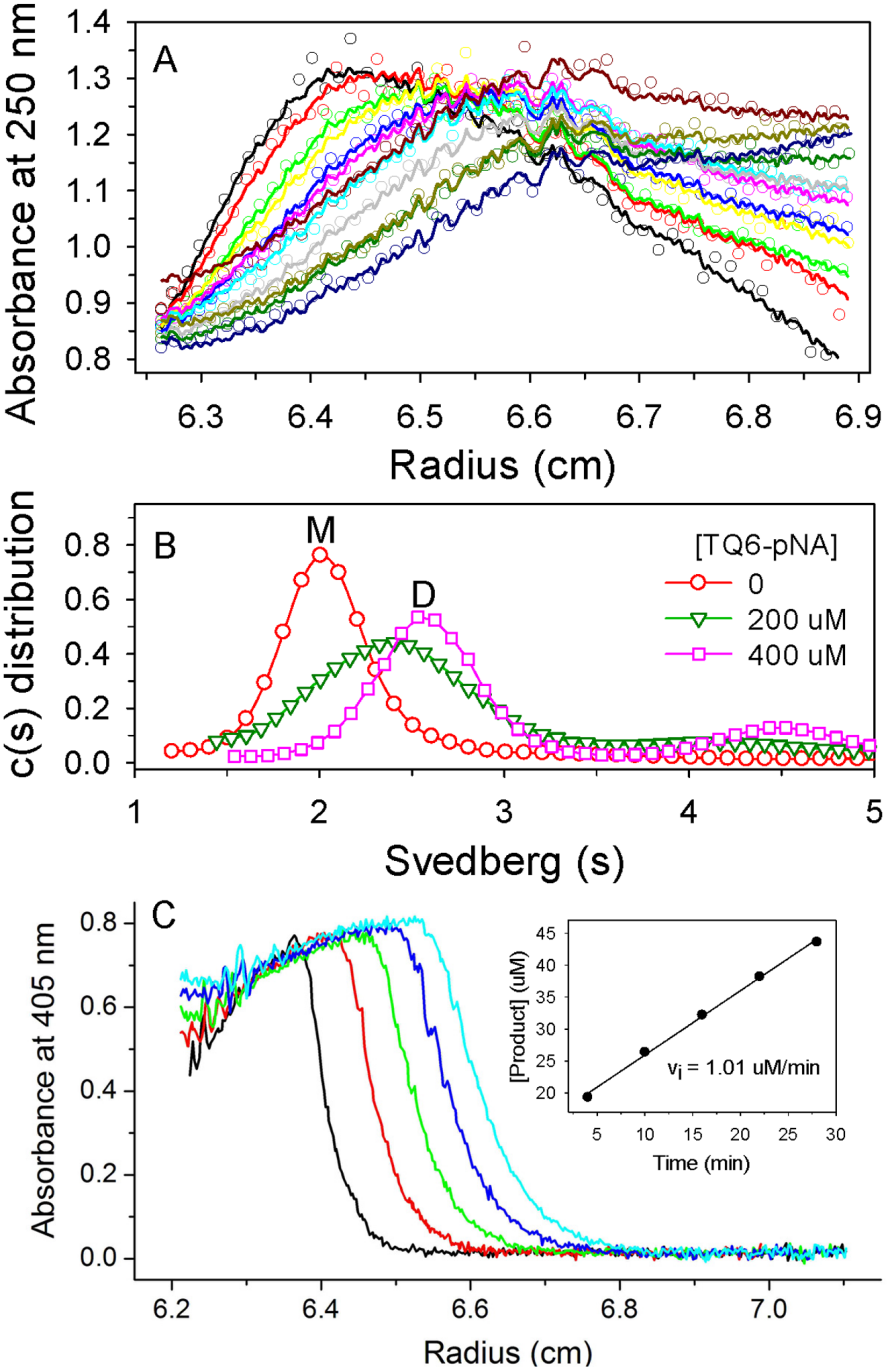
Charactering the substrate-induced dimerization of MERS-CoV M^pro^ by AEC [33]. (A) A trace of absorbance at 250 nm for the enzyme in the presence of 200 μM of TQ6-pNA during the experiment. For clarity, only every twice scan and every fifth measuring points per scan are shown. The protein of 15 μg in 10 mM phosphate (pH7.6) was used. The substrate was dissolved in D_2_O to give a higher density, which can maintain the protein band when the centrifugation begins. The symbols are experimental data and the lines are the results fitted to the Lamn equation using SEDFIT program [31]. The continuous size distribution of the best-fit result was shown in panel B colored by green. (B) Continuous c(s) distributions of MERS-CoV M^pro^ at the concentration of TQ6-pNA of 0, 200 and 400 μM. The labels M and D showed the position of the monomer and dimer species. (C) Monitoring the enzyme activity of MERS-CoV M^pro^ during the centrifugation. The same cell in panel A was followed and the absorbance at 405 nm trace for the released product (pNA) was shown. We used the spectra of the first 30 min to calculate the product concentration at different time. The inset plot showed the product versus time and the slope of the line represented the initial velocity.

However, before the centrifugation, the enzyme had been mixed with the substrate and the catalysis began, resulting in a mixture of substrate and product with enzyme. It is unable to confirm that our observation is a substrate-induced or substrate/product-induced dimerization. To solve this, a modified AUC technique, AEC [33], was utilized to detect the quaternary structure change in the absence and presence of substrate (Fig 4). Here the enzyme solution was put into the small well of band-forming centerpiece and then flowed into the substrate-containing channel when the centrifugation began. During the centrifugation, the protein layer gradually sediment and continuously met peptide substrates. Not surprisingly, there was a broad distribution between the monomer and dimer species in the presence of 200 μM peptide substrate, while a major species shifted to the dimer in 400 μM substrate (Fig 4B). It suggests that MERS-CoV M^pro^ acts as a rapid self-associated and substrate-induced dimerization. Using different strategies, Tomar *et al*. [14] confirmed that inhibitor binding can also induce and maintain the dimerization of M^pro^. On the other hand, we measured the velocity of the product formation during the centrifugation; although the rate (0.017 μM/s) is 10-fold lower than that by the spectrometric assay (Fig 4B).

To quantitatively characterize the monomer-dimer equilibrium of M^pro^ in the absence and presence of substrate, the AUC results at protein concentrations of 1.5, 6 and 15 μM were globally fitted to the monomer-dimer self-association model (Table 2). The analysis indicated that the K_d_ value of MERS-CoV M^pro^ in the absence of substrate was 7.7 μM and this decreased 11-fold in the presence of substrate, which brings it close to the value for SARS-CoV M^pro^ (1.7 μM; Table 2). Thus it can be concluded that, like the SARS-CoV M^pro^ R298A mutant [25], the presence of substrate is able to induce the dimerization of MERS-CoV M^pro^. In addition, although the K_d_ values of wild-type SARS-CoV M^pro^ without or with substrates show no significant difference (Table 2), it was possible to detect substrate-induced dimerization at a protein concentration of 1 μM by AEC [33].

Previous studies have demonstrated that a conserved residue, Glu166, plays a pivotal role in connecting the substrate binding site with the dimerization interface of SARS-CoV M^pro^ [28]. Here the equivalent residue, Glu169, was mutated and its influence on MERS-CoV M^pro^ evaluated (Fig 1B). Not surprisingly, compared to the wild-type enzyme, the activity (k_cat_) of E169A showed a 6-fold decrease (Fig 2B, open diamonds and Table 2). Furthermore, AUC analysis suggested that this enzyme consisted of a single species close to monomeric form even in the presence of substrate (Fig 3E). The K_d_ values of the E169A mutant without substrate was 14 μM, which is 2-fold higher than that of the wild-type and this did not decrease in the presence of substrate (Table 2). The results suggest that mutation of Glu169 is able to block substrate-induced dimerization and that this results in a decrease in enzyme activity. Unexpectedly, as well as the monomer, some octamer (6.3%) with a sedimentation coefficient of 4.9 S was observed in the presence of substrate (Fig 3E). Previous studies have suggested that a super-active octamer of SARS-CoV M^pro^ can be locked by 3D domain swapping [35]. In this study, the presence of the octamer form of the MERS-CoV M^pro^ E169A mutant in the presence of substrate may explain why this mutant is not totally inactive. Taking the above as a whole, the dual role of the conserved Glu residue in catalysis and dimerization is consistent for both M^pro^.

Our results confirmed that there are fewer intermolecular polar interactions at the dimerization interface of MERS-CoV M^pro^ and this results in the enzyme being in the monomer form in aqueous buffer; this contrasts with SARS-CoV M^pro^, which is mostly in the dimer form in similar circumstances due to the greater number of intermolecular interactions (Figs 1B and 3B). Based on the sequence alignment, three residues in the dimerization interface vary in coronaviral M^pro^ sequences (S1 Fig). In SARS-CoV M^pro^, residues Arg4, Ser123 and Arg298 are different to the equivalent ones in MERS-CoV M^pro^, which are Val4, Thr126 and Met298, respectively. Based on the above, three single mutants, V4R, T126S and M298R, were generated and their proteolytic activity and dimerization were assessed. Unexpectedly, both the V4R and the T126S mutant showed a higher K_d_ for the dimer to monomer and a lower level of activity than wild-type M^pro^ (Fig 2B and Table 2). K_d_ values were decreased by 1.5-fold to 2.4-fold for the two mutants in the presence of substrate, which suggests reduced substrate-induced dimerization (Fig 3C and 3D). Based on the current structure, residue Val4 showed a hydrophobic contact with another protomer’s residue Gly141 (Fig 1B). Mutation of valine to arginine may lose the contact. Furthermore, the side chain of residue Val131 of MERS-CoV M^pro^ is hydrophobic while that of the equivalent residue, Cys128 of SARS-CoV M^pro^, is hydrophilic (Fig 1B). Val131 is close to the Arg4 and will disfavor the electrostatic interaction of Arg4…Glu290 in MERS-CoV M^pro^. These variance may result in V4R failed to form a stable dimer. For the T126S mutant, after compared with the other two MERS-CoV M^pro^ structures (PDB entry 4YLU and 4WME), we found that the side chain of Thr126 is free of rotation. It is able to make a hydrophobic interaction with the residue Tyr121, resulting in a 180°-rotation of the phenol ring, and lead to a hydrogen-bond with the backbone amide of Leu144 (Fig 1B). It further results in the side chain of Leu144 toward another protomer’s Ile300 and make a hydrophobic contact for the two protomers. Mutation of Thr126 to serine will lose this contact and may disfavor the dimerization.

By way of contrast, the M298R mutant resulted in a stable dimer form in phosphate buffer (Fig 3F). The K_d_ value for the mutant in the absence and presence of substrate were 1.1 and 0.7 μM, respectively, which are very close to those for SARS-CoV M^pro^ (Table 2). Furthermore, the stable dimer form showed higher proteolytic activity and the mutation transformed the enzymes rate constant pattern at various substrate concentrations into a classical saturation curve (Fig 2C, open triangles). In addition, the K_m_ of the M298R mutant is 5.8-fold lower than that of SARS-CoV M^pro^, which suggests a higher substrate binding affinity (Table 2). It can be concluded that mutation of residue Met298 to arginine within the MERS-CoV M^pro^ results in the stabilization of the dimer formation, which in turn gives rise to more efficient catalysis. Based on our structure, Arg298 is able to make a hydrogen bonding interaction with Thr126. Residue Thr126 can be replaced by serine because the T126S/M298R double mutant also shows similar dimerization characteristics (Fig 3G) and saturated catalytic pattern to that of the M298R mutant (Fig 2C, open hexagons and Table 2). However, it can only be achieved in the presence of Arg298, not Met298.

### Substrate binding analysis of MERS-CoV M^pro^ by ITC

**Fig 5:**
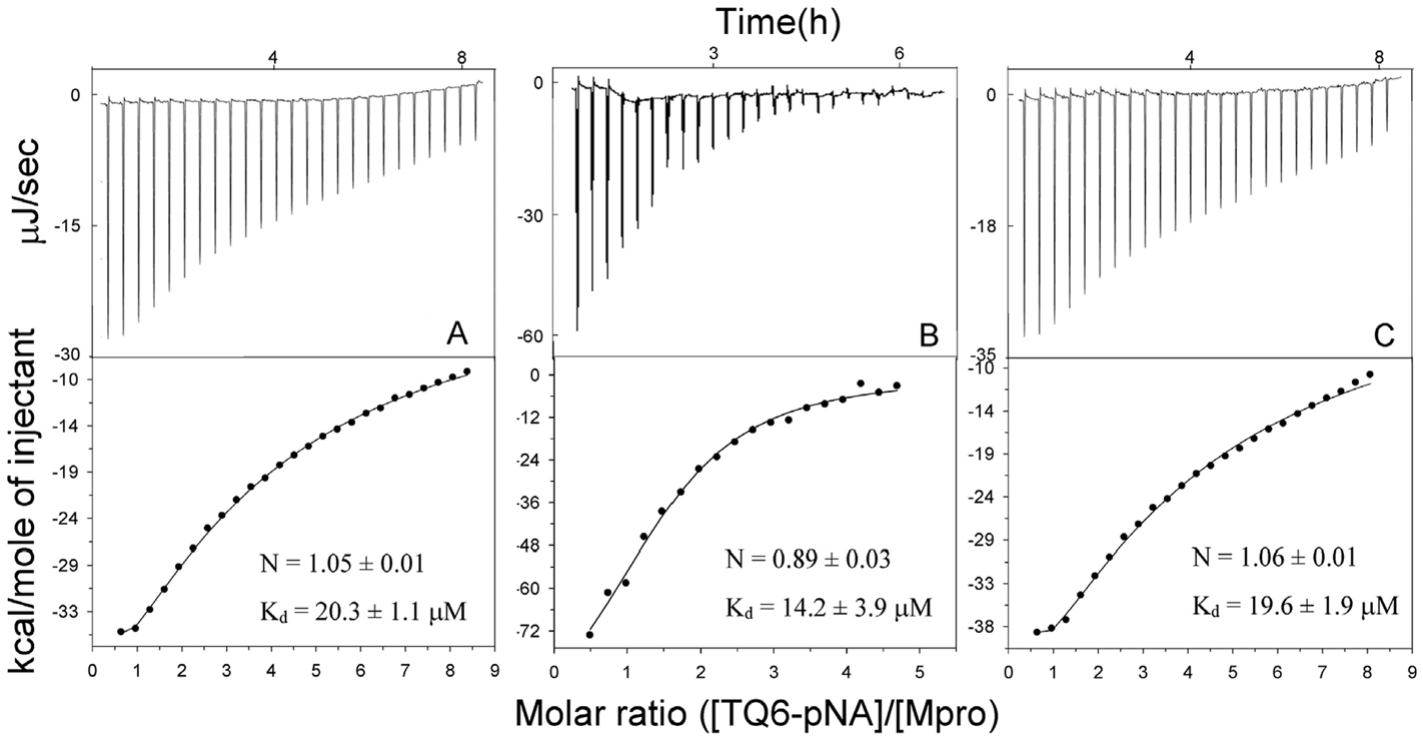
ITC titration of peptide substrate binding to MERS-CoV M^pro^ wild-type (A), E169A (B) and M298R (C) mutants. Raw data are shown in the top panel of each panel and represent the power input into the sample ampoule over time. Integrated data are shown in the bottom panels in terms of the total energy required for equilibration as a function of the molar ratio of substrate to M^pro^. The solid circles show the observed values and the lines represent the fitted results by ligand binding analysis using the Digitam program (TA instruments). The best-fit parameters for stoichiometry (N) and K_d_ are shown in the bottom panels.

The sigmoid nature of the curve describing the rate constant pattern at various substrate concentrations (Fig 2B) means that it is not possible to obtain a K_m_ value for this enzyme, which would allow us to evaluate the substrate binding affinity of the wild-type MERS-CoV M^pro^ and its mutants; the exceptions being the M298R single mutant and the T126S/M298R double mutant (Fig 2). To further delineate the binding of substrate to the enzyme, ITC was used to measure the K_d_ for the substrate (or substrate/product)-enzyme complex and the binding stoichiometry (N) (Fig 5). During the titration, the enzymatic hydrolysis might produce additional heat, resulting in higher ΔH. So we can only compared the N and K_d_ of the wild-type M^pro^ with those of E169A and M298R mutants. The three enzymes exhibited similar N (0.89 to 1.06) and K_d_ (14.2 to 20.3 μM). This suggested that the monomeric and dimeric M^pro^ show quite the same substrate binding affinity. Such phenomenon is also found in monomeric and dimeric SARS-CoV M^pro^, whose N and K_d_ for the same substrate were 0.97 to 1.05 and 29.9 to 33.8 μM, respectively [28]. With the K_m_ ((k_-1_+k_cat_)/k_1_), k_cat_ and K_d_ (k_-1_/k_1_), we are able to calculate the k_1_ and k_-1_, the rate of the association and dissociation of the enzyme-substrate complex. The k_1_ and k_-1_ for the M298R mutant and substrate is 0.049 s^-1^μM^-1^ and 0.98 s^-1^, while those for SARS-CoV M^pro^ is 0.00246 s^-1^μM^-1^ and 0.084 s^-1^. The rate constants for M298R mutant showed 20- and 12-fold higher than those for SARS-CoV M^pro^. The more rapid association and relatively slower dissociation of enzyme-substrate complex may be used to explain why the catalytic efficiency (k_cat_/K_m_) of the MERS-CoV M^pro^ M298R mutant is higher than that of SARS-CoV M^pro^ (Table 2).

## Conclusion

**Fig 6:**
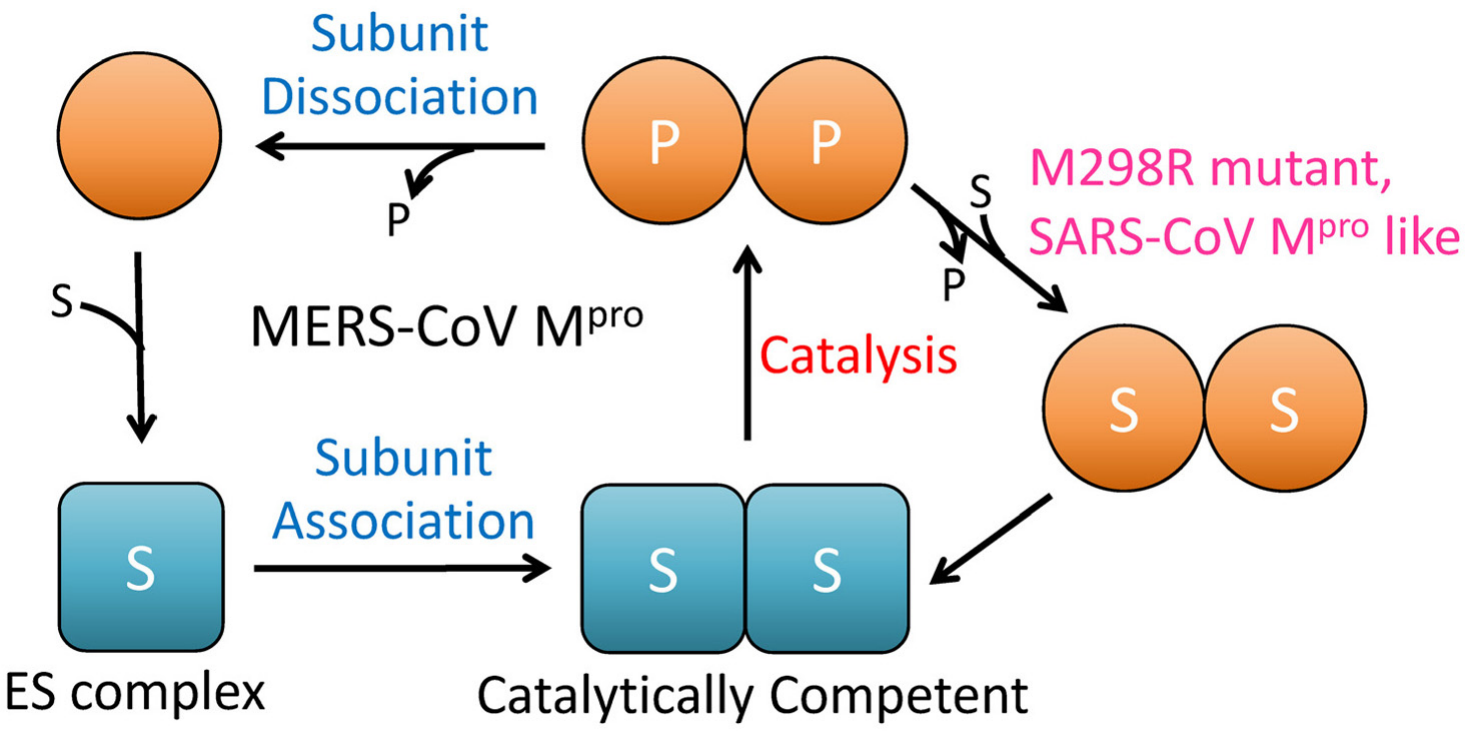
Schematic model of the catalytic process of MERS-CoV M^pro^. Substrate binding results in an active protomer (square) and then dimer formation. After a catalytic cycle, the dimer will dissociate to form the inactive protomers (circle). The M298R mutant, like SARS-CoV M^pro^, is able to maintain a dimer form without dissociation before the next round’s substrate binding and catalysis.

The crystal structure of authentic N-terminus MERS-CoV M^pro^ was determined and this was found to involve a dimeric form with less intermolecular polar interactions. Biochemical and AUC studies indicated that MERS-CoV M^pro^ shows almost the same proteolytic activity as SARS-CoV M^pro^; although it is a monomer in aqueous buffer and displays substrate-induced dimerization (Fig 6). A conserved residue, Glu169, plays an essential role in the substrate-induced dimerization of both MERS-CoV M^pro^ and SARS-CoV M^pro^. Moreover, mutation of a residue in the dimerization interface, M298R, was found to result in a more stable dimer form in aqueous buffer that had higher enzyme activity; while other two mutations, V4R and T126S, showed the reverse effect. Critical assessment of the residues important to dimerization of and catalysis by MERS-CoV M^pro^ provides valuable insights into the mechanism that controls the monomer-dimer switch of important and valuable enzyme.

## Supporting Information

S1 Fig Sequence alignment of betacoronaviral M^pro^. Modified from an output from ESPript [36]. The green ovals indicate the catalytic dyad, while the blue ovals indicate the residues making intermolecular polar contact in the dimer interface of SARS-CoV Mpro. Magenta oval indicate the residue Glu playing dual role for the dimer interface and substrate binding site. Accession numbers are as follows: MERS-CoV, NC_019843.2; Bat-CoV_HKU4, ABN_010865.1; HCoV_HKU1, NC_006577.2; MHV_A59, NP_068668.2; SARS-CoV, NP_828863.1.
(PDF)

S2 Fig Expression and purification of recombinant MERS-CoV M^pro^. Protein identification by SDS-PAGE. M: molecular marker. Lane 1–5: cytoplasmic fraction, flow-through, elute from the nickel affinity column, flow-through after 4-h’s PL^pro^ treatment and protein fraction from S-300 gel-filtration column.
(PDF)

S3 Fig Comparison with the structure of MERS-CoV M^pro^ with that of SARS-CoV M^pro^ (A) and those of ligand-bound complex, dimeric C148A mutant and bat-CoV HKU4 M^pro^ (B). (A) An overlay of the current structure of MERS-CoV M^pro^ (cyan and orange) with that of SARS-CoV (grey; PDB entry 1uk3). The red arrows show the orientation change affecting the two domain IIIs. Spheres show the two catalytic dyads. (B) Overlay of the current structure with ligand-bound complex (red; PDB entry 4YLU), dimeric C148A mutant (magenta; PDB entry 4WME) and bat-CoV HKU4 M^pro^ (yellow; PDB entry 2YNA).
(PDF)

S4 Fig Activity assay of SARS-CoV M^pro^. The plot of rate constant (k_obs_) versus the concentration of TQ6-pNA are indicated. The line represented the best-fit results according to the Michaelis-Menten equation (Eq 1). The protein concentration was 1.1 μM. The assays were performed in 10 mM phosphate (pH7.6) and repeated twice to ensure reproducibility and the error bars were shown. The kinetic parameters are shown in Table 2.
(PDF)

S5 Fig Continuous size distribution of MERS-CoV M^pro^ at various protein concentrations. The protein concentration of 1.5 (solid line), 6 (dotted line) and 15 μM (dashed line) of MERS-CoV M^pro^ were used and monitored the size distribution by AUC. The best-fit results suggest that the major species was a monomer with minor shift of sedimentation coefficient (1.74 to 1.97), while the calculated molar mass was from 35.8 to 36.6 kDa. The residual bitmaps of the raw data and the best-fit results are shown in the insets.
(PDF)

S6 Fig Effect of substrate concentration on the dimerization of MERS-CoV M^pro^. (A) Continuous c(s) distribution of the enzyme at peptidyl substrate (TQ6-pNA) concentrations of 0 μM (solid circles), 80 μM (open circles), 200 μM (solid triangles), 340 μM (open triangles), 400 μM (solid squares) and 600 μM (open squares). The protein concentration was 0.25 mg/ml. (B) Sedimentation coefficient shifts of the major species of MERS-CoV M^pro^ at different TQ6-pNA concentrations.
(PDF)

## Abbreviations

^1^AUC: analytical ultracentrifugation
AEC: active enzyme centrifugation
CoV: coronavirus
ITC: isothermal titration calorimetry
MERS-CoV: Middle East respiratory syndrome coronavirus
M^pro^: main protease
SARS-CoV: severe acute respiratory syndrome coronavirus
TQ6-pNA: TSAVLQ-para-nitroanilide
