# Supplementary figures and images for: Critical Assessment of the Important Residues Involved in the Dimerization and Catalysis of MERS Coronavirus Main Protease
Source: PLoS One. 2015 Dec 14;10(12):e0144865. doi: 10.1371/journal.pone.0144865 (PMC4682845; doi:10.1371/journal.pone.0144865)

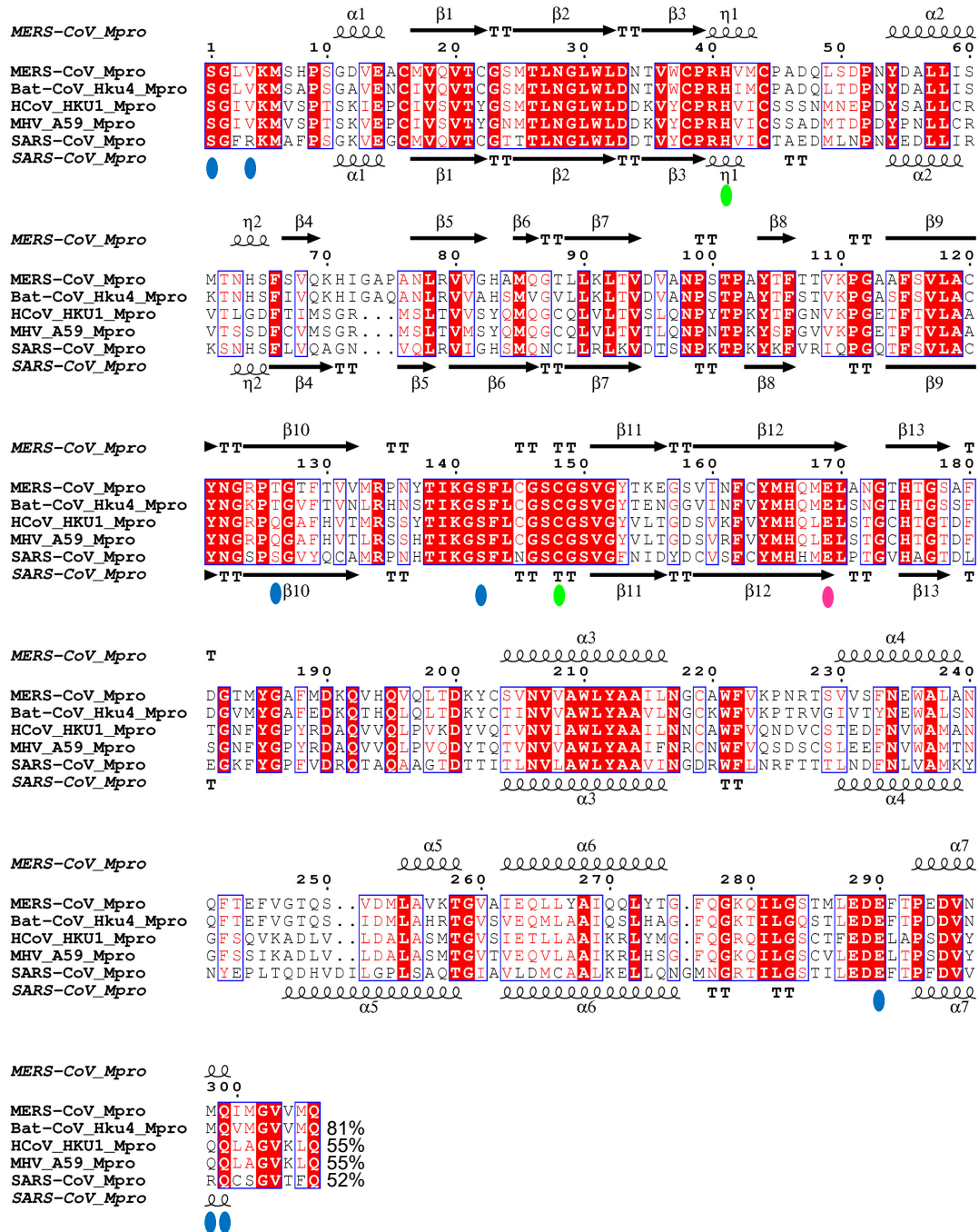

S1 Fig. Sequence alignment of betacoronaviral M<sup>pro</sup>.

Supplement: S1 Fig — Modified from an output from ESPript [36]. The green ovals indicate the catalytic dyad, while the blue ovals indicate the residues making intermolecular polar contact in the dimer interface of SARS-CoV Mpro. Magenta oval indicate the residue Glu playing dual role for the dimer interface and substrate binding site. Accession numbers are as follows: MERS-CoV, NC_019843.2; Bat-CoV_HKU4, ABN_010865.1; HCoV_HKU1, NC_006577.2; MHV_A59, NP_068668.2; SARS-CoV, NP_828863.1. (PDF) [file pone.0144865.s001.pdf]

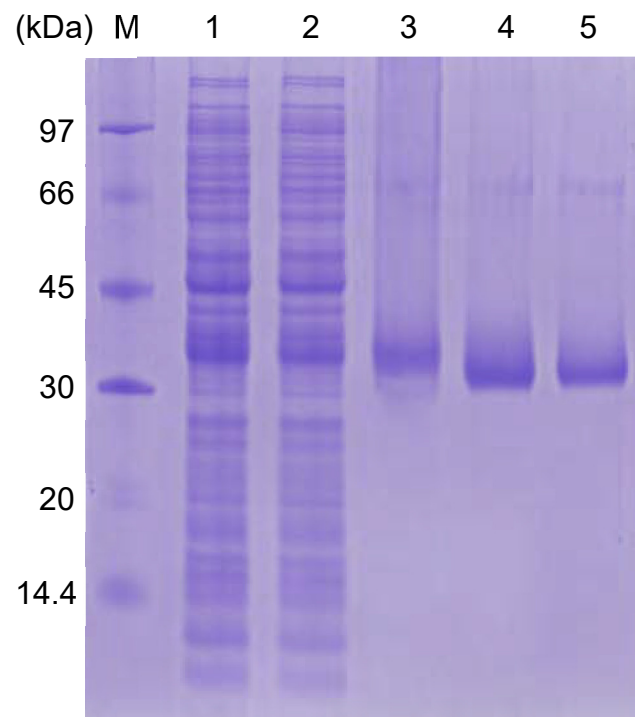

**S2 Fig. Expression and purification of recombinant MERS-CoV M<sup>pro</sup>.**

Supplement: S2 Fig — Protein identification by SDS-PAGE. M: molecular marker. Lane 1–5: cytoplasmic fraction, flow-through, elute from the nickel affinity column, flow-through after 4-h’s PLpro treatment and protein fraction from S-300 gel-filtration column. (PDF) [file pone.0144865.s002.pdf]

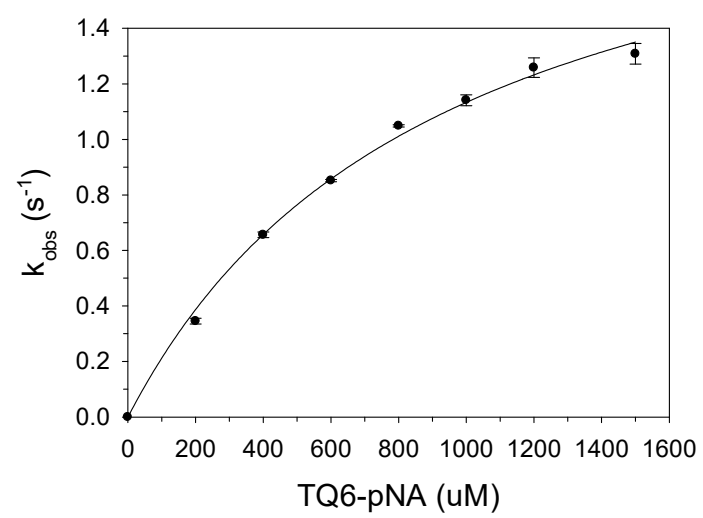

**S4 Fig. Activity assay of SARS-CoV M<sup>pro</sup>.**

Supplement: S4 Fig — The plot of rate constant (kobs) versus the concentration of TQ6-pNA are indicated. The line represented the best-fit results according to the Michaelis-Menten equation (Eq 1). The protein concentration was 1.1 μM. The assays were performed in 10 mM phosphate (pH7.6) and repeated twice to ensure reproducibility and the error bars were shown. The kinetic parameters are shown in Table 2. (PDF) [file pone.0144865.s004.pdf]

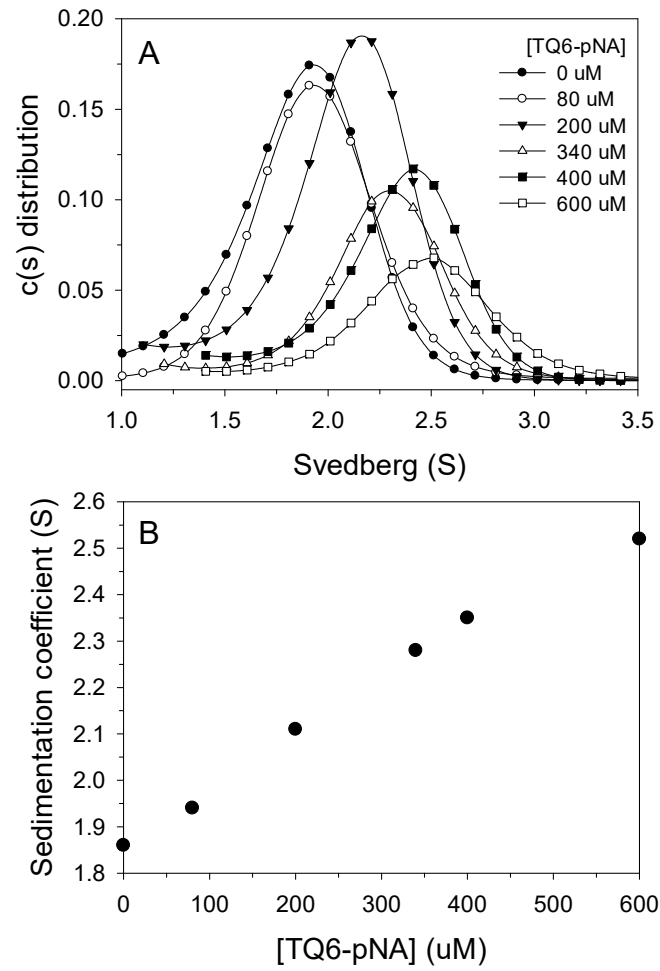

**S6 Figure. Effect of substrate concentration on the dimerization of MERS-CoV M<sup>pro</sup>.**

Supplement: S6 Fig — (A) Continuous c(s) distribution of the enzyme at peptidyl substrate (TQ6-pNA) concentrations of 0 μM (solid circles), 80 μM (open circles), 200 μM (solid triangles), 340 μM (open triangles), 400 μM (solid squares) and 600 μM (open squares). The protein concentration was 0.25 mg/ml. (B) Sedimentation coefficient shifts of the major species of MERS-CoV Mpro at different TQ6-pNA concentrations. (PDF) [file pone.0144865.s006.pdf]
