# Supplementary material for: Critical Assessment of the Important Residues Involved in the Dimerization and Catalysis of MERS Coronavirus Main Protease
Source: PLoS One. 2015 Dec 14;10(12):e0144865. doi: 10.1371/journal.pone.0144865 (PMC4682845; doi:10.1371/journal.pone.0144865)
Supplement: S3 Fig — (A) An overlay of the current structure of MERS-CoV Mpro (cyan and orange) with that of SARS-CoV (grey; PDB entry 1uk3). The red arrows show the orientation change affecting the two domain IIIs. Spheres show the two catalytic dyads. (B) Overlay of the current structure with ligand-bound complex (red; PDB entry 4YLU), dimeric C148A mutant (magenta; PDB entry 4WME) and bat-CoV HKU4 Mpro (yellow; PDB entry 2YNA). (PDF) [file pone.0144865.s003.pdf]

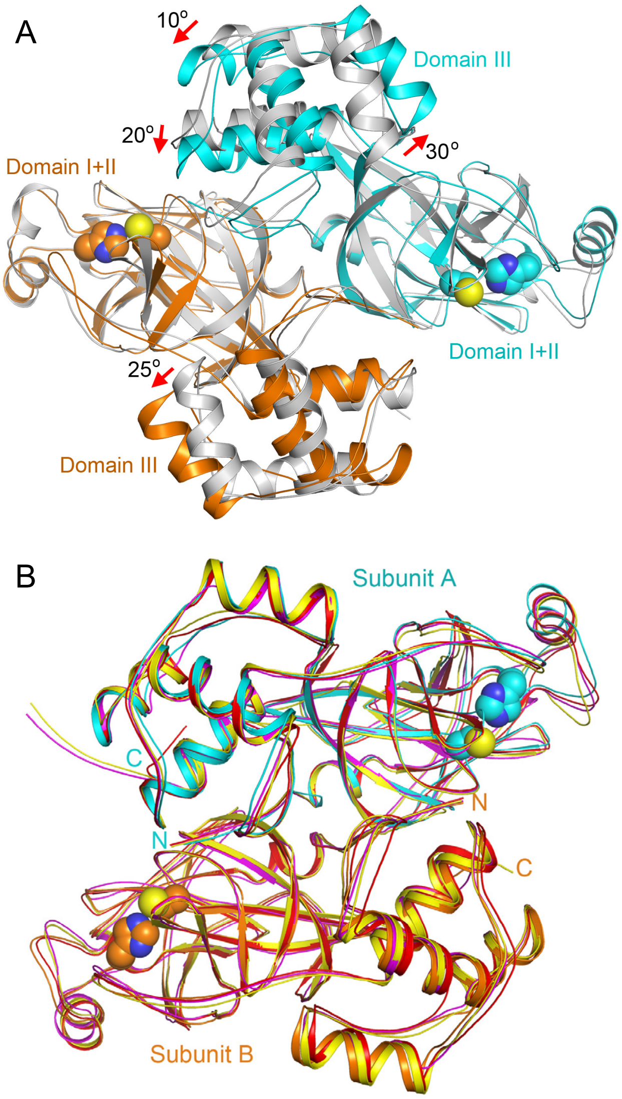

S3 Fig. Comparison with the structure of MERS-CoV M^{pro} with that of SARS-CoV M^{pro} (A) and those of ligand-bound complex, dimeric C148A mutant and bat-CoV HKU4 M^{pro} (B).
