# Supplementary material for: Critical Assessment of the Important Residues Involved in the Dimerization and Catalysis of MERS Coronavirus Main Protease
Source: PLoS One. 2015 Dec 14;10(12):e0144865. doi: 10.1371/journal.pone.0144865 (PMC4682845; doi:10.1371/journal.pone.0144865)
Supplement: S5 Fig — The protein concentration of 1.5 (solid line), 6 (dotted line) and 15 μM (dashed line) of MERS-CoV Mpro were used and monitored the size distribution by AUC. The best-fit results suggest that the major species was a monomer with minor shift of sedimentation coefficient (1.74 to 1.97), while the calculated molar mass was from 35.8 to 36.6 kDa. The residual bitmaps of the raw data and the best-fit results are shown in the insets. (PDF) [file pone.0144865.s005.pdf]

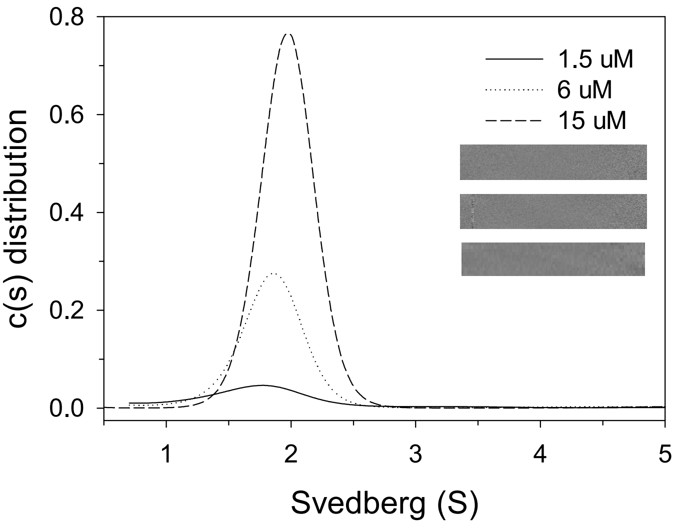

S5 Figure. Continuous size distribution of MERS-CoV M^{pro} **at various protein concentrations.**
